# A novel method to quantify perivascular space enlargement near the syrinx in a rodent model of post-traumatic syringomyelia

**DOI:** 10.1038/s41598-023-42275-y

**Published:** 2023-09-12

**Authors:** Liam Johnson, Florence Bartlett-Tomasetig, Sandra Fok, Renee Whan, Joel Berliner, Sarah J. Hemley, Marcus A. Stoodley, Lynne E. Bilston

**Affiliations:** 1 UNSW Medicine, Sydney, NSW Australia; 2 Katharina Gaus Light Microscopy Facility, Mark Wainwright Analytical Centre, UNSW, Sydney, NSW Australia; 3 Faculty of Medicine, Health and Human Sciences, Macquarie University, Sydney, NSW Australia; 4 Neuroscience Research Australia & School of Clinical Medicine, UNSW, Sydney, NSW Australia

**Keywords:** Spinal cord, Spinal cord diseases, Spinal cord diseases, Spine structure

## Abstract

Posttraumatic syringomyelia (PTS) is an enigmatic condition characterized by the development of fluid-filled cysts (syrinxes) within the spinal cord. Perivascular spaces (PVS) are a critical component of fluid transport within the central nervous system (CNS), with dilated PVSs variably implicated in the pathogenesis of syringomyelia. The extent and spatial distribution of dilated PVSs in syringomyelia, however, remains unclear. This study aims to develop a method to assess PVS dimensions across multiple spinal cord segments in rats with PTS. Syrinxes were induced in two Sprague–Dawley rats at C6/7 with computer-controlled motorized spinal cord impaction; two control rats underwent sham laminectomies. Spinal cord segments were obtained at C4, C6 and C8, cleared via tissue clearing protocols, stained with immunofluorescent antibodies and imaged under confocal microscopy. Qualitative and quantitative analyses of PVS size were performed. Arteriolar PVSs were enlarged in the perisyringeal region of the spinal cord, compared to the control cord. No PVS enlargement was observed above or below the syrinx. These results confirm previous incidental findings of enlarged PVSs in the perisyringeal region, providing new insights into PVS dimensions across multiple spinal segments, and providing a novel method for quantifying spinal cord perivascular space size distributions.

## Introduction

Perivascular, or Virchow–Robin, spaces are thought to be a major route of fluid and solute transport in the central nervous system (CNS). The precise role of PVSs in cerebrospinal fluid (CSF) circulation and solute clearance, however, remains the subject of considerable debate. Rennels et al.^1^ and Stoodley et al.^2^ first demonstrated rapid fluid influx into the CNS via PVSs in the brain and spinal cord of animal models, respectively, using subarachnoid space tracer injections. In more recent years, the ‘glymphatic’ theory proposed by Iliff et al.^3^ posits periarterial CSF influx and perivenous interstitial fluid (ISF) efflux, ascribing to brain PVSs a lymphatic-like function in interstitial waste clearance. This theory, however, remains controversial, particularly the efflux routes. Furthermore, the extent to which fluid circulation in the spinal cord mimics that in the brain is not yet clearly understood^4–9^. Better understanding of the structure of the spinal cord perivascular spaces, particularly the ability to quantify the dimensions of the perivascular network would aid in filling this knowledge gap.

PVSs have long been implicated in the pathogenesis of syringomyelia, a potentially debilitating neurological condition in which fluid-filled cysts (syrinxes) form in the spinal cord. In the case of posttraumatic syringomyelia (PTS), the syrinx forms at the level of the traumatic lesion. It occurs in approximately 28% of patients following spinal cord injury^10^. As the syrinx enlarges, it exerts increasing pressure on the surrounding neural tissue, inducing progressive sensory, motor and/or autonomic deficits^11^. Surgical management of PTS is suboptimal, and long-term deterioration is common^10^. It has been proposed that spinal arachnoid adhesions associated with spinal cord traumatic lesions disrupt CSF flow within the subarachnoid space, increasing subarachnoid space pressure and enhancing CSF flow into the cord, inducing syrinx development, however this remains to be proven^10,12,13^.

Previous observations of PVS dilatation have typically been confined to the perisyringeal region of the spinal cord and/or have been anecdotal in nature^14–16^. Few studies have attempted to systematically quantify the spatial variation in PVS structure/dimensions across multiple spinal cord levels, and none in the context of syringomyelia, and such information would potentially be informative for understanding syrinx pathogenesis. Quantitative measurements of spinal PVS are also useful for computational modelling of CNS fluid circulation, both in disorders such as syringomyelia, but also for better understanding of drug delivery to the CNS e.g. intrathecal drug delivery^17^. Comprehensive quantitative measurements PVS dimensions are not currently available.

This study aims to develop and test a method to characterize, quantify, and map the structure of the PVSs in the spinal cords of rats, in order to better understand how these spaces are altered in the context of PTS. To achieve this, control and syrinx rat spinal cords (using an established animal model of PTS) were cleared via CUBIC (Clear, Unobstructed Brain/Body Imaging Cocktails) protocols^18^, stained with immunofluorescent antibodies to vascular smooth muscle, endothelium and astrocytes, and imaged under confocal microscopy. We hypothesized that the spinal PVSs of rats with PTS would be enlarged in comparison to control spinal cords at the level of the spinal lesion. For the purposes of the current study, PVSs are defined as the spaces surrounding penetrating arterioles and venules within the CNS, bordered by the glia limitans perivascularis (astrocytic endfeet parenchymal border) and the outer border of the blood vessel wall, consistent with recent interpretations of Lam et al.^5^ and Liu et al.^19^.

## Methods

### Ethical approvals

This study complied with the relevant guidelines and regulations. The study protocol was approved by the University of New South Wales Animal Care & Ethics Committee (17/45B) and Macquarie University Animal Ethics Committee (2016/032-3). All experimentation was performed in accordance with ARRIVE guidelines.

### Animal model of post-traumatic syringomyelia (PTS)

Syrinxes were induced in two adult male Sprague–Dawley rats (4–5 weeks of age) using a computer-controlled spinal cord impactor (initial spinal cord injury at C6/C7) and subarachnoid injection of kaolin (arachnoiditis) under general anesthesia, induced with 5% isoflurane and maintained with 2–2.5% isoflurane via a nose cone. Two control rats received sham C6/C7 laminectomies, but no impact or kaolin. After 10 weeks, the rats were sacrificed via transcardiac perfusion with heparinized 0.01 M phosphate-buffered saline (PBS), and 4% paraformaldehyde. The development of this animal model, including the time for syrinx development, has been described previously^20^.

### Tissue processing and immunostaining

Spinal cords were harvested, post-fixed in 4% formaldehyde in 0.01 M PBS overnight (4 °C), washed in 0.01 M PBS (3 × 10 min) and preserved in 0.01 M PBS supplemented with 0.02% sodium azide until commencement of tissue clearing. Samples were first incubated for 2 weeks at 37 °C (100 rpm) with CUBIC reagent L [10 wt% Triton X-100, 10 wt% *N*-buthyldiethanolamine, Water], then washed in 0.01 M PBS at 37 °C, 100 rpm (3 × 6 h). Samples were freshly embedded in 2% low melting agarose gel and cut via vibratome into successive 100 μm section (8 sections taken above the syrinx, 8 at the level of the syrinx and 8 below the syrinx, approximating spinal segments C4, C6 and C8 respectively). Samples were washed in 0.01 M PBS (3 × 10 min) and incubated with primary antibodies, 1:100 anti-GFAP (Sigma-Aldrich, Cat. No. SAB2500462) and 1:100 anti-PECAM-1 (Santa Cruz Biotechnology, Cat. No. sc-1506, Abcam), in 0.5% BSA for 2 days at 37 °C, under gentle agitation. Samples were subsequently washed in 0.01 M PBS (3 × 10 min), prior to secondary antibody staining with 1:100 Cy2 (Jackson ImmunoResearch, Cat. No. 705-225-147), 1:100 anti-SMA-Cy3 (anti-SMA-Cy3) (Sigma-Aldrich, Cat. No. C6198) and 1:100 Cy5 (Jackson ImmunoResearch, Cat. No. 711-175-152), in 0.5% BSA for 2 days at 37 °C, under gentle agitation and wrapped in foil. After washing in 0.01 M PBS (3 × 10 min), samples were immersed in CUBIC-reagent R [45 wt% antipyrine, 30 wt% nicotinamide, Water] at 37 °C (100 rpm) for 3 days prior to confocal microscopy.

### Imaging

For quantitative measurements of PVS sizes, samples were imaged with confocal microscopy (Zeiss LSM 800) with 10× and 40× objectives. Laser wavelengths 488 nm, 561 nm and 640 nm were used for collecting sequentially fluorescence from Cy2, Cy3 and Cy5 fluorophores, respectively. At each level, 3 to 5 of the 8 sections cut from above, below and within the syrinx region, were randomly selected and imaged at 10×/0.45 (Plan-Apochromat M27 Air) magnification first, in order to provide a broad overview of the spinal cord section (multiple tiles). For each selected section, 10 to 25 images of PVSs in association with arteries and veins were taken at 40×/1.3 magnification (Plan-Apochromat Oil DIC (UV) VIS-IR M27). Only PVSs in association with arteries were included for quantitative analysis, due to the non-specific staining of anti-PECAM-1 rendering it difficult to accurately measure perivenous space dimensions.

Image analysis. To characterize PVSs, blood vessels were segmented in a semi-automatic process, using custom software (see below) written in Java. Image regions and boundaries were detected automatically, however user intervention was used for quality control in selecting/deselecting appropriate structures.

### Region segmentation

**Figure 1:**
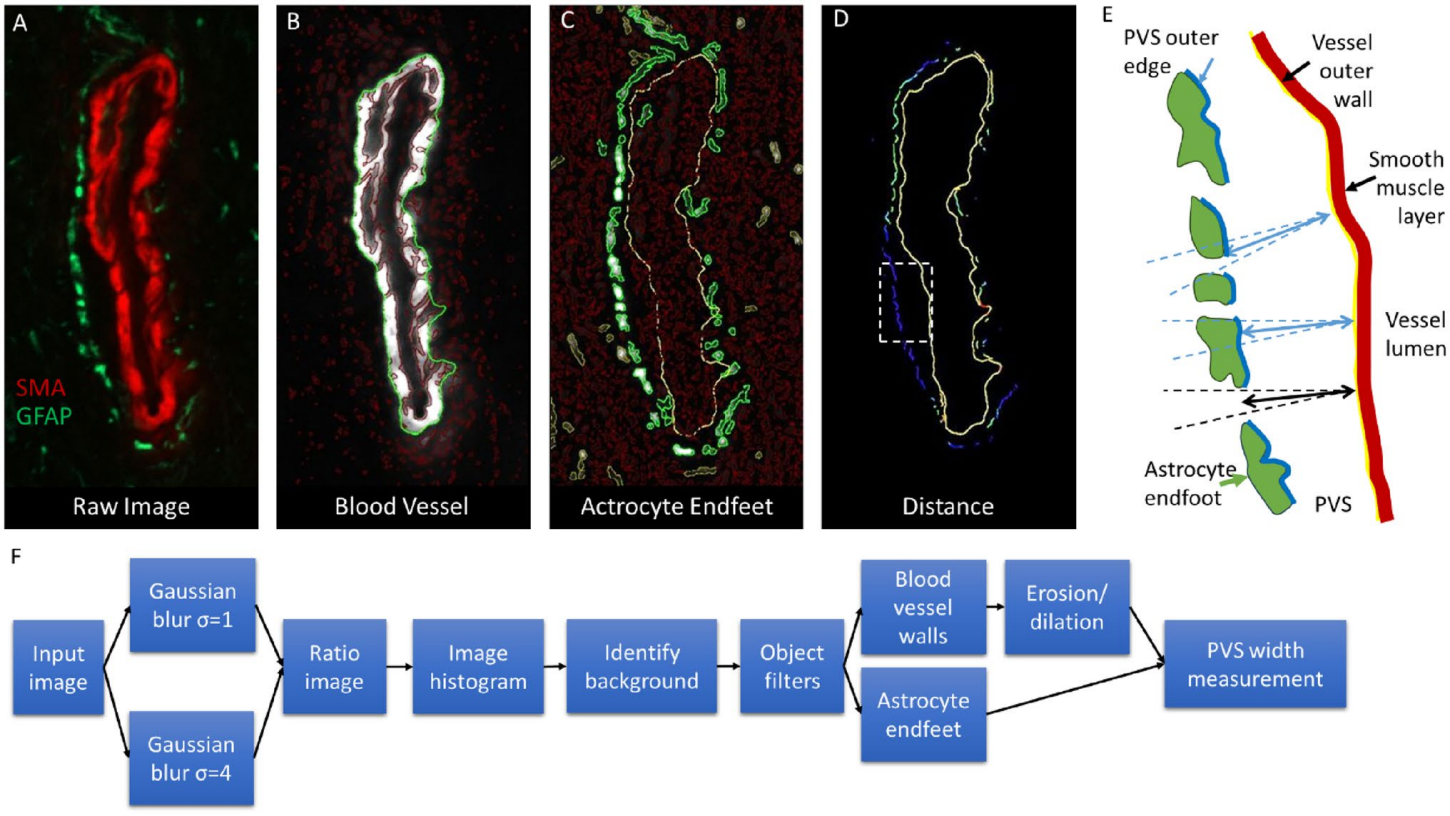
Segmentation and measurement of the perivascular space (PVS) around a sample arteriole. (**A**) Raw image showing arteriole wall stained with SMA (red) and astrocyte endfeet bordering the PVS with GFAP (green). (**B**) Segmented vessel wall (red outline around white SMA + vessel wall) and outer border with gaps filled (green line). (**C**) Astrocytic endfeet segmented (green line around white GFAP + regions). (**D**) Distance between outer vessel border (yellow line) and inner surface of surrounding astrocyte endfeet, indicated as a line color-coded with distance between the astrocytic endfoot and the arterial wall. Starting from red representing 0 µm distance, transitioning through hues to green representing 0.78 µm and then into blue representing boundaries ≥ 1.56 µm. (**E**) Schematic of the segmented regions and how the outer wall of the PVS (thick blue line) is detected using a perpendicular line (solid light blue line) from the vessel outer wall (yellow line) and two adjacent lines at 10° from normal (dashed lines). Where an astrocyte endfoot is not detected (black solid and dashed lines do not intersect an endfoot), no PVS width is calculated for that position on the vessel wall. (**F**) Flow chart showing the image processing steps used to process the confocal images to obtain the blood vessel wall (shown in **B**) and the astrocyte endfeet (shown in **C**) before calculating the PVS width (**D,E**).

A ratio image representing how pixel values change with regard to an increasing blur radius was constructed by dividing the original image with Gaussian blur σ = 1 by the original image with gaussian blur σ = 4. A ratio of 1 signifies no local change (likely background), larger values signify locally bright objects, and lower values signify locally dark objects. Inspecting the image histogram and identifying the most prominent peak identifies a value strongly associated with background. Moving to the right of this peak until the frequency count drops below 77% of the background peak frequency yielded a robust threshold for identifying all bright objects. Filters were constructed at the object level to identify genuine objects based on object area, average intensity change at object boundary, and average object intensity. Although these filters were reasonably robust, they were manually adjusted for each image if required to improve accuracy. For blood vessels, gaps in the vessel perimeter were closed using binary dilation and erosion operations. These selections are demonstrated on a sample arteriole image in Fig. 1. A diagram of the identified objects is shown in Fig. 1E, along with a flow chart demonstrating the image processing process (Fig. 1F).

### Region selection

Blood vessel regions were manually unselected if the image quality in the region was too poor for reliable segmentation. GFAP stained astrocytes at the outer PVS border were automatically selected if their closest perimeter pixel was within 2.5 µm of a blood vessel perimeter. In cases where the PVS was greater than 2.5 µm, GFAP positive objects were included through manual selection.

### PVS width measurements

Distance measurements were made around the perimeter of the arteriole. To only include the edges facing the PVS, 3 lines were projected for each pixel along the perimeter, one normal to the perimeter, and the other two were ± 10° from the normal angle. If any line intersected a segmented astrocytic endfoot, it was deemed a facing edge and assigned a distance value based on a distance map constructed from all imaged blood vessels. A sample distance map around a blood vessel is shown in Fig. 1D, and a schematic of the measurement process is shown in Fig. 1E. PVS width measurements from the syrinx region were performed by manually outlining the area in a tiled overview image for each section. Using stage metadata coordinates from the images a coarse alignment was automatically performed to align higher resolution scans to the tiled lower resolution overview. This was then manually aligned further to account for sample movement on the stage holder. After converting all regions to real world coordinates the closest distance was automatically measured for each PVS with regards to the syrinx site, or central canal for controls.

### Data analysis and statistics

For statistical analysis, PVS width measurements were averaged for each vessel, and the vessels were grouped into three regions—above, at, and below the level of the syrinx. In the controls, the equivalent spinal levels were chosen to match the spinal levels of the syrinx animals. For each vessel, the widths were categorized into quintiles based on the PVS width (nearest 20%, 20–40th%, 40–60th%, 60–80th%, and 80–100%) and the average within each quintile recorded. Data for all vessels at a given spinal level from the two animals in each group (controls, syrinx) were not different (p > 0.4 for all levels) and were pooled for subsequent analyses. Mean PVS width for all vessels at each spinal level were compared using two-way ANOVA (group, level) with a Tukey post-hoc test. Distributions of PVS width across the quintiles between controls and syrinx groups were compared using mixed models to account for repeated measures within each animal, with Sidak’s post-hoc tests to adjust for multiple comparisons. Statistical analysis was performed in SPSS (v26, IBM Statistics) and Graphpad Prism (v9.0.2, Graphpad Software Inc.).

## Results

**Figure 2:**
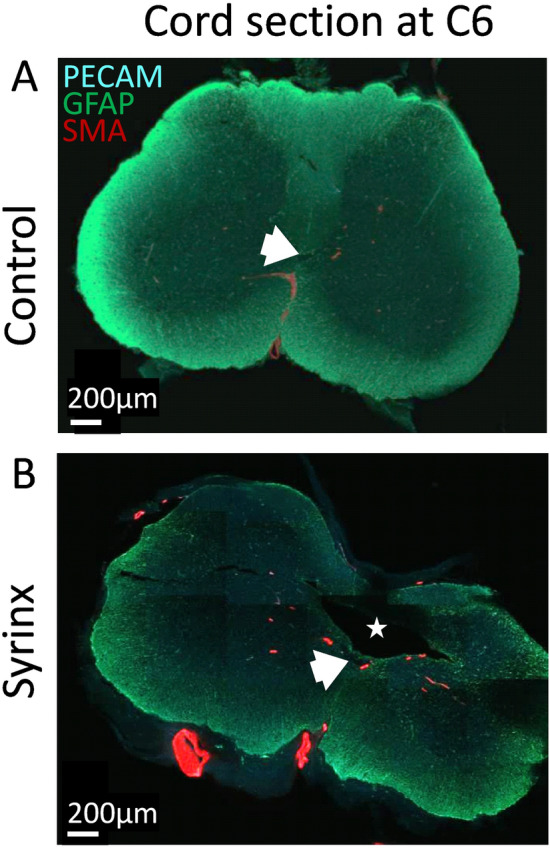
Representative confocal images of whole spinal cord sections at C6 in control (**A**) and syrinx (**B**) animals. Red immunofluorescence is anti-SMA-Cy3, green anti-GFAP/Cy2 and blue anti-PECAM-1/Cy5. The star indicates the syrinx cavity in the syrinx animal, which occurs at the site of compression from the impactor. The white block arrow indicates the central canal. The images have been brightened to assist with visualization.

**Figure 3:**
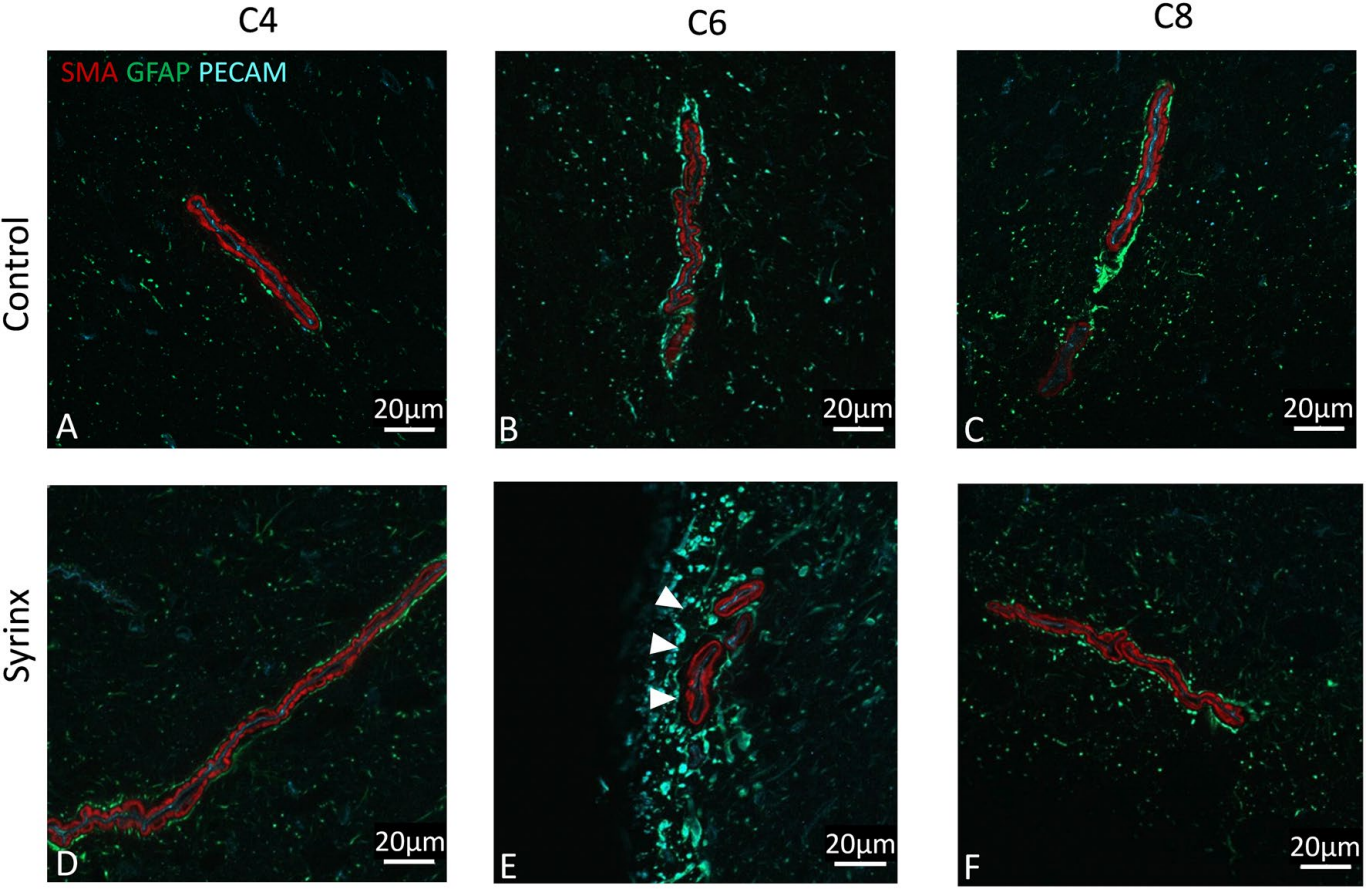
Representative confocal images of arteries in control (**A–C**) and syrinx (**D–F**) cords, at C4 (**A,D**), C6 (**B,E**) and C8 (**C,F**). The syrinx occurred at C6/7. Red immunofluorescence is anti-SMA-Cy3, green anti-GFAP/Cy2 and blue anti-PECAM-1/Cy5. In controls, the PVS appear narrow and closely follow the contour of the arterioles. This is similar in the syrinx animals above and below the syrinx, but at the level of the syrinx, the PVSs appear wider and more irregular (white arrowheads in **E**).

Confocal microscopy images of the syrinx and control cords demonstrated strong anti- SMA-Cy3, widespread anti-GFAP/Cy2 and anti-PECAM-1/Cy5 fluorescence signal throughout the axial sections, indicated by the blue-green appearance of the cord sections in Fig. 2, and more clearly visualised as separate structures in the higher magnitude images in Fig. 3. Throughout both the control and syrinx cords, few penetrating arteries (although multiple veins) were identified in the white matter, with the majority of arteries clustered around the ventral median sulcus and central canal, as indicated by the red anti-SMA-Cy3 signal visible in the sections in Fig. 2A,B, and the localisation around arterioles shown in Fig. 3. A syrinx was observed posterior to the central canal throughout the C6 section of the syrinx in both syrinx cords, and the area of spinal cord compression was clearly observable (Fig. 2).

**Figure 4:**
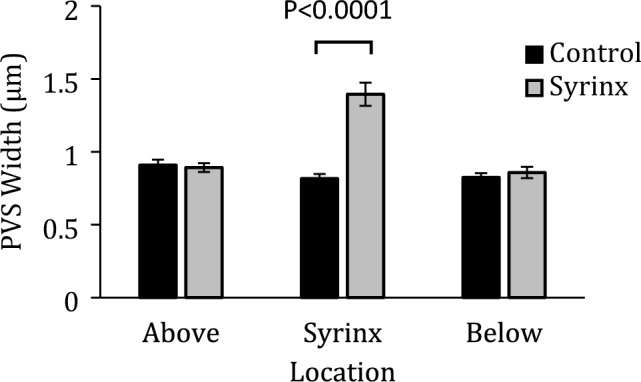
Average PVS widths for control and syrinx animals at matching spinal levels above the syrinx (C4), at the level of the syrinx (C6), and below the syrinx (C8). Error bars are standard error of the mean. Two-way ANOVA indicates that there are significant differences in mean PVS width between the control and syrinx animals (p < 0.0001), and a Tukey Post-hoc test confirms this difference is significant at the level of the syrinx (p < 0.001).

PVSs were moderately dilated in the perisyringeal region (Fig. 3E, as quantified in Fig. 4, p < 0.0001), and variably dilated further out from the syrinx. PVSs did not appear dilated in the regions above and below the syrinx (Fig. 3D,F). Quantification appears in Fig. 4). We observed considerable non-specific PECAM staining in the spinal cord parenchyma at the level of the syrinx, consistent with previous observations in spinal cord injury^21^.

**Figure 5:**
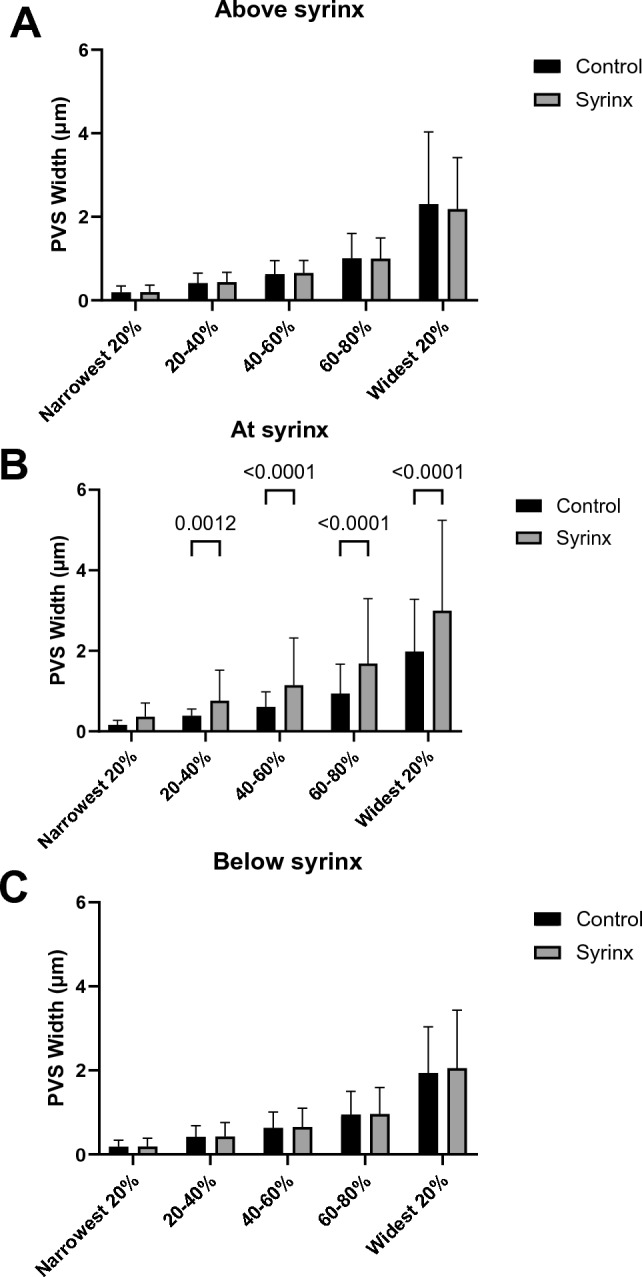
Mean PVS width in each quintile group for control and syrinx animals at each level, above the syrinx (**A**), at the syrinx (**B**) and below the syrinx (**C**). Mixed model analysis indicated that there were no differences between controls and syrinx animals either above or below the syrinx, but at the level of the syrinx, the syrinx animals had a larger mean PVS (p < 0.001 for group) width, consistent with the whole cord section data in Fig. 4. However, there was a significant interaction between quintiles and group (p < 0.001) at the level of the syrinx, indicating that the difference between groups varies across the width distribution, with the difference in PVS widths being greater amongst the wider PVSs. Sidak multiple comparisons showed significant differences between each quintile group at the level of the syrinx (B), except for the narrowest 20% of PVSs (p = 0.203).

Analysis of the PVS widths in the different locations, where the widths were grouped into quintiles indicated that at the level of the syrinx, the mean PVS width in each of the quintile groups was greater in the syrinx animals with Sidak’s post-hoc comparisons being significant for all except the narrowest quintile (see Fig. 5). There were no differences at the other levels. Quantitative data for PVS widths are in Table 1.

**Table 1:** Quantitative summary data for PVS widths for control and syrinx animals above, at, and below the level of the syrinx.

	Control			Syrinx		
	Above	Syrinx	Below	Above	Syrinx	Below
Whole section (Mean ± SEM)	0.91 ± 0.037	0.82 ± 0.031	0.82 ± 0.036	0.89 ± 0.03	0.48 ± 0.08	0.86 ± 0.039
Q1 (mean ± SD)	0.19 ± 0.16	0.16 ± 0.12	0.18 ± 0.16	0.19 ± 0.17	0.37 ± 0.34	0.19 ± 0.20
Q2 (mean ± SD)	0.41 ± 0.24	0.39 ± 0.17	0.42 ± 0.26	0.43 ± 0.24	0.76 ± 0.76	0.43 ± 0.33
Q3 (mean ± SD)	0.62 ± 0.33	0.61 ± 0.37	0.63 ± 0.38	0.65 ± 0.30	1.15 ± 1.18	0.65 ± 0.45
Q4 (mean ± SD)	1.00 ± 0.59	0.94 ± 0.73	0.95 ± 0.55	1.00 ± 0.50	1.69 ± 1.61	0.96 ± 0.63
Q5 (mean ± SD)	2.30 ± 1.73	1.98 ± 1.30	1.94 ± 1.10	2.18 ± 1.24	3.00 ± 2.25	2.05 ± 1.38

Data are means and SEM (for whole cord sections) or SD, for quintiles. Q1 is the narrowest 20% of PVSs, Q2 is 20–40%, Q3 is 40–60%, Q4 is 60–80% and Q5 is the widest 20% of PVSs.

## Discussion

Using a novel semi-automated quantitative approach, we have demonstrated that we can quantify the width of the perivascular spaces in the spinal cord of rodents in an animal model of PTS. The results of this study indicate that the PVSs around arterioles are moderately dilated in the vicinity of the syrinx compared to controls, and that this was confined to the syrinx region, since PVS dilation was not detected two spinal levels above or below the syrinx. Furthermore, this dilation occurred in PVSs across all but the narrowest quintile of PVSs at the syrinx level, suggesting almost the whole cord at the syrinx level was affected, rather than only a subset of PVSs nearest the syrinx being enlarged. In both syrinx and control cords, the PVSs were irregular in shape and tortuous, consistent with the paths of the arterioles they surround. Arteriolar PVSs were observed predominantly within the spinal cord grey matter, a finding supported by Liu et al.^19^, who noted fewer arterioles within the rat spinal cord white than grey matter.

The observed enlargement of perisyringeal PVSs near the syrinx is consistent with previous qualitative clinical and incidental observations of PVS dilatation^14,16^. In 1972, Ball and Dayan reported marked dilatation of PVSs in the perisyringeal regions of human necropsy specimens^14^. Durward et al.^16^ reported similar dilatations in the spinal cords of patients with PTS. Klekamp et al.^22^, in feline models of arachnoiditis (but not syringomyelia), observed significantly dilated PVSs in spinal cord regions adjacent to arachnoid scarring. These enlarged PVSs may explain the preferential tracer flow into a syrinx via PVSs reported by Brodbelt et al.^23^ in rat models of PTS. This is consistent with observations in humans by Heiss et al.^24^, utilising CT myelography, demonstrating increased CSF influx into syrinxes associated with subarachnoid space obstruction (spinal trauma, focal arachnoiditis, arachnoid cysts) and Chiari I malformation. More recently, Berliner et al.^25^ demonstrated increased CSF tracer penetration in spinal cord tissue post extradural constriction surgery (obstructing the subarachnoid space), in both arteriolar and venular PVSs. Together these studies suggest that increased fluid inflow is associated with a broad range of mechanisms of CSF flow obstruction, and enlarged PVSs might facilitate such flow enhancement, although cause and effect remain to be determined.

This study has several limitations which need to be kept in mind when interpreting the data. First, the tissue preparation, particularly fixation, may alter tissue structure, including the dimensions of the PVSs. Mestre et al.^26^ reported dramatic reductions in the cross sectional area of PVSs surrounding cortical surface arteries after fixation and removing the dura, suggesting that the absolute PVS dimensions reported here may be underestimates of in vivo sizes. Indeed, their average in vivo PVS width was approximately 40 µm, substantially larger than the largest PVS widths measured in our study. However, it remains unclear whether this effect could differentially affect PVSs at different locations in the spinal cord, but it seems unlikely that this would account for the relative increase in perisyringeal PVS size only at the level of the syrinx compared to control cords.

We observed extensive non-specific staining of anti-PECAM-1 in the cord parenchyma, particularly adjacent to the syrinx, which precluded reliable analysis of venous PVS dimensions. Such staining is consistent with previous studies of spinal cord injury^21^, although the exact causes are yet to be elucidated. Additional research is required to find a robust method for quantifying perivenous space dimensions. This study’s power to detect statistically significant differences in PVS dimensions is limited by its sample size (i.e. 2 syrinx and 2 control rats), increasing risk of effect size overestimation. Future studies should therefore aim to utilize a larger sample. Finally, an inherent limitation of any animal model is its applicability to human pathology. This study utilizes an established model of PTS, one that induces syrinxes with a high degree of histological similarity to human syrinxes, minimal neurological deficit and a contusion injury thought to accurately simulate human spinal cord pathology^20^.

## Conclusions

In conclusion, this study assessed PVS size and spatial distribution across multiple spinal segments in the spinal cords of rats with PTS. Our results demonstrate enlargement of arteriolar PVSs in the perisyringeal region, confirming previous clinical and incidental findings of dilated perisyringeal PVSs. No PVS enlargement was observed in segments two vertebral levels rostral or caudal to the syrinx. This study therefore provides further evidence for the potential role of dilated PVSs in the pathogenesis of PTS. The precise mechanisms underlying syrinx development, however, remain unclear and require further investigation. Future research should attempt to optimize tissue clearing and staining protocols for 3D characterisation of the spinal PVS structures, using light sheet volumetric analysis of whole cord PVS networks. This would aid computer modelling of fluid flow and further our understanding of the role of PVSs in syrinx development.

## Data Availability

Data will be made available upon reasonable request to the corresponding author.
